# Prognostic impact of mutation profiling in patients with stage II and III colon cancer

**DOI:** 10.1038/srep24310

**Published:** 2016-04-14

**Authors:** Yinchen Shen, Xiaohong Han, Jianfei Wang, Shuai Wang, Hongying Yang, Shih-Hsin Lu, Yuankai Shi

**Affiliations:** 1 Department of Medical Oncology, Beijing Key Laboratory of Clinical Study on Anticancer Molecular Targeted Drugs, National Cancer Center/Cancer Hospital, Chinese Academy of Medical Sciences and Peking Union Medical College, Beijing, China; 2 Department of Pathology, National Cancer Center/Cancer Hospital, Chinese Academy of Medical Sciences and Peking Union Medical College, Beijing, China; 3 Department of Etiology and Carcinogenesis, State Key Laboratory of Molecular Oncology, National Cancer Center/Cancer Hospital, Chinese Academy of Medical Sciences and Peking Union Medical College, Beijing, China

## Abstract

Development of colorectal cancer (CRC) associates with accumulation of genetic mutations include the epidermal growth factor receptor (*EGFR*) signaling pathway. However, whether mutations in *KRAS* together with downstream factors *BRAF*, *PIK3CA* and *NRAS* impact prognosis is still unclear for stage II-III colon cancer. In the present study a total of 228 stage II-III colon cancer samples were retrospectively collected, *KRAS* (codons 12, 13 and 61), *BRAF* (exon 11 and exon 15), *PIK3CA* (exon 9 and exon 20) and *NRAS* (codons 12, 13 and 61) status was detected by Sanger sequencing, 37.89% (86/227) tumors harbored a *KRAS* mutation, 7.02% (16/228) harbored a *BRAF* mutation, 13.18% (29/220) harbored a *PIK3CA* mutation and 0.89% (2/224) harbored a *NRAS* mutation. *NRAS* mutations existed only in stage II colon cancer. Older groups harbored a higher *KRAS* and *BRAF* mutation (P < 0.05), *PIK3CA* (exon9) mutations appeared more common in worse differentiation tumors (P = 0.032). Moreover, *PIK3CA* (E545K) mutation was significantly associated with tumor recurrence (P = 0.031) and acted independently prognostic for poor OS (P = 0.044), while only in stage III colon cancer. *KRAS*, *BRAF* and *NRAS* mutations do not have major prognostic value in stage II and III colon cancer, subtypes of gene mutations should be further investigated for a better understanding in CRC.

Colorectal cancer (CRC) is ranking the third most common cancer worldwide and the fourth leading cause of cancer-related death, which makes CRC a heavy burden in many countries and districts, including Asia^1^,^2^. Since the precision medicine initiated this year, complex diseases such as cancer and diabetes seem to start a new Odyssey^3^,^4^. Targeted therapy has already achieved great progress in many kinds of cancers included CRC, the monoclonal antibody (MoAb) cetuximab or panitumumab which targeted on epidermal growth factor receptor (*EGFR*) prolonged survival of metastatic CRC (mCRC) patients^5^, although only selected groups may benefit from these agents. However, patients with stage II or III colon cancer experienced obstacles with targeted therapy, no matter anti-*EGFR* or anti-VEGF MoAbs, which both did not appear effective when added to chemotherapy^6^,^7^,^8^.

Although *KRAS* mutation status test is recommended before targeted therapy selection^9^, the prognostic value of *KRAS* in early stage of colon cancer remains controversial. Previous studies are inconsistent in the impact of *KRAS* mutation on recurrence and death for patients with chemotherapy in early stage colon cancer^10^,^11^,^12^,^13^. *BRAF* gene consists one of the RAF gene family, which encodes a serine/threonine protein kinase belonging to the RAS-RAF-MEK pathway regulated by activated *KRAS*^14^,^15^. Generally, *KRAS* and *BRAF* mutations are mutually exclusive, moreover, *BRAF* indicates poorer prognosis for mCRC patients treated with anti-*EGFR* therapy^16^, while the role of *BRAF* in other cancer stage is still unclear, reported studies did not sustain accordance about this issue^17^,^18^,^19^,^20^. Since a myriad of *PIK3CA*-AKT pathway aberrations have been observed in human cancers, gain-of-function mutations of this gene appeared in many cancer types included CRC^21^,^22^. As the main effector in the downstream of *EGFR* intracellular signaling pathway, *PIK3CA* mutation has appeared a negative effect in MoAbs treatment in mCRC patients^16^. However, there exists limited data on mutant *PIK3CA* associated with prognostic value for stage II or III cancers. *NRAS* is closely to *KRAS*, which also a member of RAS gene^23^, although *NRAS* gene mutation is rare in CRC, approximately 2.2–4.19%^16^,^22^,^24^, it appears a valuable prognostic biomarker in anti-*EGFR* MoAbs therapy for mCRC patients^5^,^25^, then *NRAS* status for indicating prognosis should be further confirmed in early stage cancers.

The tumorigenesis and development of CRC is a multistep process with different genetic mutation accumulation^26^, driver mutations included somatic changes in *KRAS*, *BRAF*, *PIK3CA* and *NRAS* represent the main aspect in genetic alternations for CRC^27^. To date, major of the presented data were derived from western populations and few data was available for the Chinese. Herein we design this study to investigate the mutation spectrum in stage II/III colon cancer and the potential correlations with clinicopathological characteristics, furthermore, survival data of patients treated with 5-fluorouracil (5-FU)/oxaliplatin-based chemotherapy was collected, which may provide an appropriate insight between gene mutation and survival status in Chinese populations.

## Results

### Frequency and association with tumor mutations

In the cohort of 228 colon cancer patients, 124 stage II and 104 stage III tumor specimens were collected for gene mutation analysis. *KRAS* (codons 12, 13 and 61) mutants were found in 37.89% (86/227) cases, 26.87% (61/227) in codon 12, 6.61% (15/227) in codon 13 and 3.54% (8/226) in codon 61. The corresponding order of *KRAS* codon 12 mutation was G12D, G12V, G12C, G12S and G12A. For codon 13, 93.3% (14/15) mutations were G13D, and followed by G13S. Codon 13 mutations were more frequent in stage II colon cancers, with a significant association compared to stage III tumors (10.5% vs. 1.9%, P = 0.013). Q61H was the most frequent mutant subtype in codon 61, and Q61L together with Q61R were also observed in this study.

The status of *BRAF* (exon 11 and exon 15) mutations was detected in all samples, 7.02% (16/228) tumors harbored a *BRAF* mutation, 1.75% (4/228) in exon 11 and 5.26% (12/228) in exon 15. V600E was the major subtype in *BRAF* mutation (43.75%, 7/16), which appeared only in *KRAS* wild type (P = 0.046), while *BRAF* mutation was not mutually exclusive with *KRAS* mutation, for exon 11 or exon 15 (V600M) could be found in *KRAS* mutant tumors.

*PIK3CA* (exon 9 and exon 20) mutations could not be assigned to 3.5% (8/228) samples, 13.18% (29/220) harbored a *PIK3CA* mutation, 10.45% (23/220) in exon 9 and 2.7% (6/221) in exon 20. E545K mutation in exon 9 was more frequent than other subtypes, followed by E542K and E545G, we also found E545D, Q546R and Q546K mutation in these samples. The H1047R consisted 83.3% (5/6) mutations in exon 20, and only one specimen harbored a H1047Y mutation. *PIK3CA* mutation appeared a strong association with *KRAS* mutation, 21.69% (18/83) in mutants versus 8.09% (11/136) in wild types (P = 0.005). However, only exon 9 mutation had the association (P = 0.022) and exon 20 did not share this (P = 0.202).

*NRAS* (codons 12, 13 and 61) mutations existed only in stage II colon cancer, 0.89% (2/224) tumors harbored a *NRAS* mutation, of which were G12D in codon 12 and Q61R in codon 61. The rare mutants in *NRAS* did not yield any significant association with other mutants. Besides, we also found that *NRAS* and other gene mutations were mutually exclusive in this cohort.

### Clinicopathological characteristics of *KRAS*/*BRAF*/*PIK3CA*/*NRAS* gene mutations

In the univariate analysis, both *KRAS* and *BRAF* mutations were more frequent in older patients (P < 0.05), and female patients shared higher *KRAS* mutations (codon 12 and codon 61) frequency, meanwhile patients with tumor family history showed more *KRAS* (G12D) mutations (30.4% vs. 11.8%, P = 0.023) and stage II tumors harbored a higher *KRAS* codon 13 mutations (10.5% vs. 1.9%, P = 0.013). Moreover, with N stage accelerated, *KRAS* codon 13 mutations declined (P < 0.05). We found *BRAF* mutants only existed in non-smokers (P = 0.046) and no other significant differences were discovered when compared to wild type. As for *PIK3CA* mutations, no significant association appeared between gene mutants and the characteristics, while subgroup mutants such as*PIK3CA* (exon9) mutations appeared more common in worse differentiation tumors (P = 0.032), and exon 20 mutations did not share this (P = 0.593). Besides, exon 9 (E545K) mutation was significantly associated with tumor recurrence (10.7% vs. 0.0%, P = 0.031) in stage III patients, but not for stage II or combined together. *NRAS* mutations were too rare to attain significant associations with any clinicopathological characteristics in this study (Table 1).

### Univariate analysis of outcomes predictors

**Figure 1:**
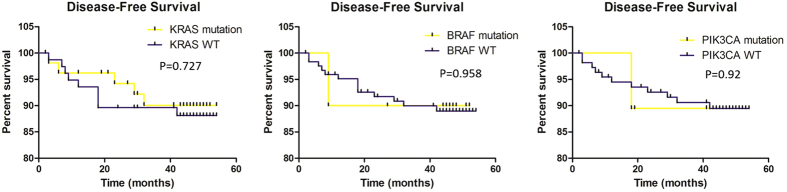
Associations between gene mutations and disease-free survival (DFS). No significant associations existed. *P* value for *KRAS*/*BRAF*/*PIK3CA* gene were 0.727, 0.958 and 0.92, respectively. WT: wild type.

**Figure 2:**
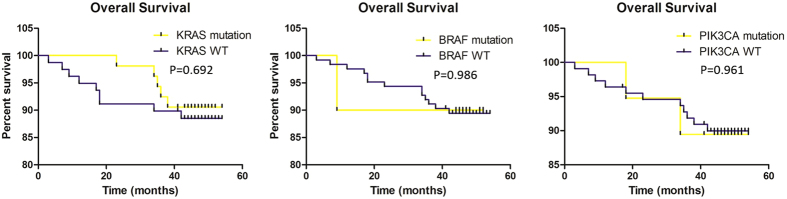
Associations between gene mutations and over-all survival (OS). No significant associations existed. *P* value for *KRAS*/*BRAF*/*PIK3CA* gene were 0.692, 0.986 and 0.961, respectively. WT: wild type.

The median follow-up for outcomes analysis in this cohort was 46.0 months, 55.9% (66/118) stage II and 84.9% (79/93) stage III patients had received 5-FU/ oxaliplatin-based standard chemotherapy after surgery, consequent treatment of the other 17 patients were unclear. Only patients who received adjuvant chemotherapy after surgery were recruited in the final analysis and none of them had accepted radiotherapy or anti-*EGFR*/*VEGF* MoAbs targeted therapy. No significant difference in DFS was observed between patients with *KRAS* wild type and mutants (P = 0.727), furthermore subgroups such as codon 12/13 and mutation types (G12D/G12V/G12C/G12S/G13D) did not appear as prognostic factors for DFS, stratified by tumor stage or analyzed together, we also failed to discover significant associations between *BRAF*/*PIK3CA*/*NRAS* mutations and DFS (Fig. 1). However, *KRAS* codon 61 and *PIK3CA* (E545K) mutations were prognostic for DFS in stage III alone, but not in stage II or the whole population. *KRAS* mutation status was not prognostic for OS, no matter analyzed together or separately. Other gene mutations also did not obtain significance with OS (Fig. 2), while similarly *KRAS* codon 61 and *PIK3CA* (E545K) mutants showed prognostic for OS (Table 2). We also discovered that tumor differentiation had significant relationship with DFS and OS, in patients for stage III and combined together (P < 0.05).

### Multivariate analysis of outcome predictors

In the multivariate analysis, we selected sex, age, tumor differentiation, nodal stage, *KRAS*, *BRAF* and *PIK3CA* gene mutations in relation to DFS and OS by stage and in both stages combined together. The *KRAS* codon 61 mutation still achieved significance after adjustment, which acted as an independent prognostic factor for DFS and OS in stage III alone (P = 0.026 and 0.018 respectively). However, *PIK3CA* (E545K) mutant status was prognostic for OS (P = 0.044), while a significant trend for DFS only in stage III (P = 0.051). Other gene mutations still failed to obtain significance for DFS or OS in the multivariate analysis (mutant subtypes and tumor stage involved). The interaction emerged for DFS and OS with tumor differentiation still exited in patients for stage III and combined together (P < 0.05) (Table 3).

## Discussion

In this retrospective study, we evaluated common hot spot gene mutations located downstream of *EGFR* signaling pathway, the potential prognostic value for *KRAS*, *BRAF*, *PIK3CA* and *NRAS* was assessed in 228 stage II-III colon cancer. Since anti-*EGFR* MoAbs improved survival in mCRC patients based on specific RAS gene mutations^5^,^28^, biomarkers such as gene mutations or amplifications has sparked thriving researches for outcomes prognosis. As for stage II-III colon cancer, *KRAS* and *BRAF* gene mutations were evaluated frequently in different studies, however, no consistence was observed^10^,^11^,^12^,^29^. Gene mutation profiling alters for different ethnics was confirmed in previous studies^22^,^30^,^31^, then potential prognostic value of gene mutations should be reconsidered in Chinese populations.

Our data presented here demonstrated that *PIK3CA* (E545K) mutation was significantly associated with tumor recurrence in stage III, besides, E545K also played as an independent prognostic gene biomarker for adverse outcomes in stage III colon cancer alone. Although *KRAS* codon 61 appeared significant association with DFS and OS for stage III cancer, while only one patient died with mutant *KRAS* codon 61 tumor, considering the rare death event in this group, the magnitude of *KRAS* codon 61 status for prognosis was confused, which should be investigated further in the future. We did not discover any other gene mutant had impact for DFS or OS on stage II-III colon cancer patients treated with 5-FU/oxaliplatin-based standard chemotherapy, *KRAS* tumor mutation status has no major prognostic value for survival was consistent with a previous analysis in a large cohort, though *BRAF* mutations achieved significance for OS in their study^11^. Associations between *KRAS* or *BRAF* mutation and worse outcomes were reported in North Central Cancer Treatment Group N0147 trial, in which prognostic impact of *KRAS* and *BRAF* mutants were involved with stage III colon cancer patients received FOLFOX with or without cetuximab^32^. The adverse effects of *KRAS* or *BRAF* mutation in stage II/III colon cancer was not observed in this study, which may be due to the small sample size and consequent lower mutant tumors. However, considering the different treatment strategy in these studies (i.e. FU/FA alone or combined with irinotecan^11^), comparison of the prognostic value of these biomarkers should be more careful. Previous studies which involved Eastern Asia populations showed *KRAS* mutation was associated with worse prognosis in stage III or high-risk stage II colon cancer patients^10^, while lack of significant association between *BRAF* mutation and DFS was in accordance with our results and other clinical trial researches based on western populations^11^,^12^,^33^. Different populations and treatment regime may be responsible for the final outcomes, we should also notice that diverse therapies were performed after tumor relapse, the over-all survival of patients would be influenced in more aspects. To what extent the *KRAS*/*BRAF* gene mutations impact on survival in stage II/III colon patients should be further confirmed.

We found *PIK3CA* (E545K) was prognostic for poor OS independently in stage III colon cancers, the adverse effect of *PIK3CA* in early stage colon cancers were previously reported^34^,^35^, although other studies presented no associations between *PIK3CA* mutation and survival^36^. Reasons for the differences may include different target populations, covariates studied in the Cox model, diverse drugs usage and the methodologies for mutations detection. *PIK3CA* mutation (exon9) significantly associated with *KRAS* mutant tumors, which was consistent with reported results^16^,^22^,^37^, the exon9 mutations were highly dependent on RAS-GTP binding, especially in E542K and E545K^38^, which may suggest that *PIK3CA* mutations occur after *KRAS* mutations, in a later step during the tumorigenesis of CRC^39^. E545K also related to the tumor recurrence in stage III cancer patients, all the three E545K mutations appeared only in relapse tumors (10.7% vs. 0.0%, P = 0.031), while E545K was rare in this cohort because of the small sample size, and which consisted a relative small proportion (23.1%, 3/13) in *PIK3CA* exon9 mutants, then precise conclusions on the prognostic value of E545K should be investigated more in the future. *NRAS* mutations were only 0.89% (2/224) in this cohort, which was obviously lower than reported results (2.9–4.0%)^13^,^36^, the different detection method and study population may explain the distinct *NRAS* mutant frequency. We did not find significant associations between *NRAS* mutation and patients’ survival, which was consistent with previous studies^12^,^13^,^36^. In multivariate analysis, tumor differentiation appeared independently prognostic value in colon cancer patients for stage III and combined together, poor differentiation indicated more malignant tumors, and the results were in accordance with a recent study^40^, though the tumor differentiation acted as prognostic for survival only in univariate analysis in their study.

The frequencies of mutations in *KRAS*, *BRAF*, *PIK3CA* and *NRAS* were compared with previous researches, the *KRAS* and *BRAF* mutation frequencies were close to early studies^11^,^12^,^18^,^41^, however, data based on another population presented lower *KRAS* and *BRAF*mutants^10^, more sigmoid and rectum tumors involved in their cohort may contribute to the lower mutation frequency. Older patients harbored a higher gene mutation showed aberration accumulation with aging^12^. The *PIK3CA* mutation (13.18%) was lower than some previous data, which ranged from 12.2 to 20.18%^12^,^35^,^36^,^42^,^43^,^44^, ethnic population, various detection methods and whether rectum cancer involved may explain the difference, the *PIK3CA* mutation did not significantly associated with clinicopathological characteristics, which was consistent with reported results^35^,^43^,^44^. However, subtype of *PIK3CA* mutation may impact the tumor recurrence and survival, because few E545K mutants in our cohort, the relationship involved in this analysis may be not robust, then further studies or pool analysis of reported researches may focus on this. *NRAS* mutations were only 0.89% in present study, while another study based on high sensitive detection method showed a 2.9% mutation frequency^12^, and both studies did not discover any correlation between *NRAS* mutation and clinicopathological characteristics or patients’ survival. Considering rare data provided for *NRAS* mutations in stage II/III colon cancer, large population cohort investigation for this gene mutation detection should be performed, as *NRAS* closely related to *KRAS* and *NRAS* mutation was prognostic for *EGFR* MoAbs therapy inmCRC^28^. Several limitations were included in present study such as relatively small sample size and limited information of rare gene mutants to support confirmed conclusions. Though KRAS codon 61 appeared as prognostic biomarker in stage III cancer, the rare event may indicate indeterminacy at present, considering the low codon 61 mutation frequency in CRC^22^,^27^, confirmation of the prognostic value required further investigation with a larger sample size. Additionally, other potentially biomarkers for prognosis were not evaluated, which contained DNA mismatch repair (MMR) and microsatellite instability (MSI) status, or the CpG island methylator phenotype (CIMP). Reported studies provided the relationship between survival and these biomarkers, though the conclusions were still controversial^10^,^12^,^18^,^32^,^35^. We should strive to obtain more precise and simpler genome aberrations for prognosis, even in cancers marked as a complicated multistep disease, more investigations should be implemented to bring us a step closer to personalized medicine in the future.

In conclusion, we conducted this study to describe the gene mutation profiling in stage II/III colon cancer in Eastern Asia populations, *PIK3CA* (E545K) mutation was independently prognostic for OS in stage III and different subtypes of gene mutants should be further investigated in colon cancers, additional analysis with these molecular prognostic factors may provide a better insight and help us understand the mutation profiling in the evolution of this disease.

## Methods

### Patients

We totally recruited 124 stage II and 104 stage III colon cancer patients who received complete resection of tumor at the Cancer Hospital of the Chinese Academy of Medical Sciences (Beijing, China) between September 2010 and October 2011. None of them was treated with chemotherapy or radiotherapy previously and each of them was contacted to provide formalin-fixed, paraffin-embedded (FFPE) tumor tissues. Disease-free survival (DFS) was defined as time from operation to the first confirmed relapse, or alive without recurrence at last contact, and time to death or last follow-up from surgery was defined as overall survival (OS). The patients’ demographic and clinicopathological data are presented in Table 1.

### Ethics statement

The experimental protocol in this study was approved by the Institutional Review Board (IRB) in Cancer Hospital of Chinese Academy of Medical Sciences and Peking Union Medical College, and informed consent was obtained from all patients. All experiments were carried out in accordance with relevant guidelines and regulations.

### DNA isolation and mutation assessment

Hematoxylin and eosin (HE) staining was implemented to guarantee specimens tested contained >80% cancer cells, and areas enriched malignant cells were identified before DNA extraction by two independent pathologists. DNA was extracted by the QIAamp DNA FFPE Tissue Kit (Qiagen, Hilden, Germany) according to the manufacturer’s instructions and stored at −80 °C until use.

Mutation hotspots in *KRAS* (codons 12 and 13), *BRAF* (exon15), *PIK3CA* (exon 9 and exon 20) and *NRAS* (codon 61) were detected, moreover, rare types of mutants for *KRAS* (codon 61 in exon 3), *BRAF* (exon 11) and *NRAS* (codons 12 and 13) were also included. We performed the polymerase chain reaction (PCR) amplification as followed for *KRAS*, *BRAF*, *NRAS* and *PIK3CA* exon 20: 1 min of initial denaturation at 95 °C, 35 cycles of amplification consisting of 30 s at 94 °C, 40 s at 57 °C, and 30 s at 72 °C, with a final additional elongation at 72 °C for 7 min. *PIK3CA* exon 9 amplification was carried out with a touchdown PCR program: 94 °C (2 min); 3 cycles of 94 °C (30 sec), 64 °C (30 sec), 70 °C (30 sec); 3 cycles of 94 °C (30 sec), 61 °C (30 sec), 70 °C (30 sec); 3 cycles of 94 °C (30 sec), 58 °C (30 sec), 70 °C (30 sec); 32 cycles of 94 °C for (30 sec), 57 °C (30 sec), 70 °C (40 sec); 1 cycle of 70 °C (5 min). Non-template control was included in each batch during the experiment, sequencing was applied using ABI 3500xL Genetic Analyzer (Applied Biosystems, Carlsbad, CA, USA).

### Statistical analysis

Univariate relationships between gene mutation status and clinicopathological characteristics were performed by the Chi-square (χ2) test. Multivariate relationships were evaluated by fitting logistic regression analysis, using a backward stepwise (likelihood ratio) method and variables which showed statistically significant association with gene mutations were subjected to final regression analysis. The clinical database was last updated in March 2015. DFS and OS were done with the Kaplan-Meier survival function with the method of log-rank test, the hazard ratios were calculated with Cox proportional hazard regression models. All statistical tests were two-sided and the significance level was set at *P* < 0.05. Data analysis was performed by the SPSS 17.0 statistical software (SPSS, Inc., Chicago, IL, USA).

## Additional Information

**How to cite this article**: Shen, Y.C. *et al*. Prognostic impact of mutation profiling in patients with stage II and III colon cancer. *Sci. Rep*. **6**, 24310; doi: 10.1038/srep24310 (2016).

## Figures and Tables

**Table 1:** Characteristics of 228 colon cancer patients and association of gene mutations with clinicopathological parameters.

Characteristics	Number (%)	*KRAS*		*BRAF*		PI3KCA		*NRAS*	
		Mutations (%)	*P*	Mutations (%)	*P*	Mutations (%)	*P*	Mutations (%)	*P*
Sex									
Male	137 (60.1)	49 (36.0)	0.489	8 (5.8)	0.434	18 (13.5)	0.849	0 (0)	0.157
Female	91 (39.9)	37 (40.7)		8 (8.8)		11 (12.6)		2 (2.2)	
Age									
≤60	111 (48.7)	34 (30.9)	0.041	4 (3.6)	0.049	13 (12.1)	0.694	1 (0.9)	0.96
>60	117 (51.3)	52 (44.4)		12 (10.3)		16 (14.2)		1 (0.9)	
Mean	60 ± 12								
Range	23-84								
Tumor family history									
Yes	23 (10.2)	9 (40.9)	0.82	0 (0)	0.382	6 (26.1)	0.09	0 (0)	1.0
No	203 (89.0)	77 (37.9)		16 (7.9)		22 (11.3)		2 (1.0)	
Missing	2 (0.8)								
Tumor location*									
Right	107 (46.9)	43 (40.2)	0.584	7 (6.5)	0.792	18 (17.1)	0.097	1 (0.9)	1.0
Left	121 (53.1)	43 (35.8)		9 (7.4)		11 (9.6)		1 (0.8)	
Tumor differentiation									
Well /Moderate	191 (84.1)	69 (36.3)	0.356	13 (6.8)	0.724	21 (11.5)	0.104	2 (1.1)	1.0
Poor	36 (15.5)	16 (44.4)		3 (8.3)		8 (22.2)		0 (0)	
Missing data	1 (0.4)								
Tumor stage↑									
II	124 (54.4)	48 (38.7)	0.779	8 (6.5)	0.715	14 (11.8)	0.5	2 (1.7)	0.5
III	104 (45.6)	38 (36.9)		8 (7.7)		15 (14.9)		0 (0)	
Depth of invasion↑									
T2	7 (3.1)	3 (42.9)	1.0	0 (0)	1.0	0 (9.7)	0.122	0 (0.0)	1.0
T3	207 (91.2)	78 (37.9)		15 (7.2)		25 (12.6)		2 (1.0)	
T4	13 (5.3)	5 (38.5)		1 (7.7)		4 (30.8		0 (0.0)	
Missing data	1 (0.4)								
Nodal stage↑									
N0	124 (54.4)	49 (39.5)	0.62	8 (6.5)	0.94	14 (11.8)	0.768	2 (1.7)	1.0
N1	58 (25.5)	19 (32.8)		5 (8.6)		8 (14.5)		0 (0.0)	
N2	45 (19.7)	18 (40.9)		3 (6.7)		7 (15.6)		0 (0.0)	
Missing data	1 (0.4)								
Relapse									
Yes	35 (15.3)	14 (41.2)	0.699	3 (8.6)	0.725	4 (11.4)	1.0	0 (0.0)	1.0
No	170 (74.6)	64 (37.6)		12 (7.1)		22 (13.6)		2 (1.2)	
Missing data	23 (10.1)								

Tumor location*: Right is defined as right colon and transverse colon; left is defined as left colon and sigmoid colon.

↑: Seventh edition of the AJCC/UICC TNM staging systems.

**Table 2:** Univariate analysis of outcome predictors.

		DFS			OS			
Stage/Gene	Mutant Number	*P*	Hazard Ratio	95% CI	*P*	Hazard Ratio	95% CI	
*KRAS*								
Stage II	28	0.389	2.749	0.249 to 30.324	0.400	2.802	0.254 to 30.911	
Stage III	25	0.412	0.574	0.152 to 2.163	0.404	0.568	0.151 to 2.144	
Stage II and III	53	0.727	0.824	0.276 to 2.458	0.692	0.802	0.269 to 2.393	
*BRAF*								
Stage II	6*	–	–	–	–	–	–	
Stage III	4	0.578	1.792	0.229 to 14.030	0.563	1.833	0.234 to 14.336	
Stage II and III	10	0.958	0.946	0.124 to 7.235	0.986	0.982	0.128 to 7.510	
*PIK3CA*								
Stage II	8*	–	–	–	–	–	–	
Stage III	11	0.945	1.056	0.228 to 4.891	0.904	1.099	0.237 to 5.086	
Stage II and III	19	0.920	0.926	0.207 to 4.140	0.961	0.964	0.216 to 4.306	

*No death events in this group.

**Table 3:** Multivariate analysis of outcome predictors.

Prognostic marker	Stage II (n = 66)			Stage III (n = 79)			Stage II/III (n = 145)		
DFS	P	Hazard Ratio	95% CI	P	Hazard Ratio	95% CI	P	Hazard Ratio	95% CI
Sex	0.978	278790.681	0.0001 to ∞	0.464	1.701	0.411 to7.0	0.352	1.869	0.501 to 9.0
Age	0.419	2.729	0.239 to 31.2	0.152	3.199	0.652 to 15.7	0.069	3.305	0.91 to 12.0
Tumor differentiation	0.217	5.049	0.386 to 66.1	0.022	3.879	1.215 to 12.4	0.007	4.264	1.486 to 12.2
Nodal stage	–	–	–	0.895	1.086	0.317 to 3.7	0.142	1.675	0.841 to 3.3
*KRAS* mutation	0.542	2.234	0.169 to 29.5	0.372	0.543	0.142 to 2.1	0.702	0.807	0.269 to 2.4
*BRAF* mutation	0.990	<0.0001	0.0001 to ∞	0.927	1.103	0.136 to 8.9	0.762	0.729	0.094 to 5.7
*PIK3CA* mutation	0.964	<0.0001	0.0001 to ∞	0.383	0.461	0.081 to 2.6	0.368	0.48	0.097 to 2.4
OS									
Sex	0.978	285332.546	0.0001 to ∞	0.462	1.697	0.415 to 6.9	0.339	1.904	0.508 to 7.1
Age	0.454	2.537	0.222 to 28.9	0.112	3.623	0.742 to 17.7	0.050	3.59	0.98 to 12.9
Tumor differentiation	0.190	5.486	0.429 to 70.1	0.029	3.642	1.14 to 11.6	0.006	4.358	1.526 to 12.4
Nodal stage	–	–	–	0.934	1.054	0.305 to 3.6	0.170	1.618	0.813 to 3.2
*KRAS* mutation	0.540	2.234	0.171 to 29.2	0.334	0.514	0.133 to 2.0	0.567	0.725	0.24 to 2.2
*BRAF* mutation	0.990	<0.0001	0.0001 to ∞	0.992	0.989	0.121 to 8.1	0.652	0.622	0.079 to 4.9
*PIK3CA* mutation	0.963	<0.0001	0.0001 to ∞	0.430	0.496	0.087 to 2.8	0.427	0.523	0.105 to 2.6
